# Next‐generation sequencing in two cases of *de novo* acute basophilic leukaemia

**DOI:** 10.1111/jcmm.16591

**Published:** 2021-06-16

**Authors:** Takuya Shimizu, Tadakazu Kondo, Yasuhito Nannya, Mizuki Watanabe, Toshio Kitawaki, Takero Shindo, Masakatsu Hishizawa, Kouhei Yamashita, Seishi Ogawa, Akifumi Takaori‐Kondo

**Affiliations:** ^1^ Department of Hematology and Oncology Graduate School of Medicine Kyoto University Kyoto Japan; ^2^ Department of Pathology and Tumor Biology Graduate School of Medicine Kyoto University Kyoto Japan; ^3^ Department of Hematology Kyoto‐Katsura Hospital Kyoto Japan

**Keywords:** acute basophilic leukaemia, gemtuzumab ozogamicin, next‐generation sequencing

## Abstract

Acute basophilic leukaemia (ABL) is a rare subtype of acute myeloid leukaemia (AML); therefore, few data are available about its biology. Herein, we analysed two ABL patients using flow cytometry and next‐generation sequencing (NGS). Two cell populations were detected by flow cytometry in both patients. In Case no. 1, blasts (CD34^+^, CD203c^−^, CD117^+^, CD123dim^+^) and basophils (CD34^−^, CD203c^+^, CD117^±^, CD123^+^) were identified, both of which were found by NGS to harbour the 17p deletion and have loss of heterozygosity of *TP53*. In Case no. 2, blasts (CD33^+^, CD34^+^, CD123^−^) and basophils (CD33^+^, CD34^+^, CD123^+^) were identified. NGS detected *NPM1* mutations in either blasts or basophils, and *TET2* in both. These data suggest an overlap of the mutational landscape of ABL and AML, including *TP53* and *TET2* mutations. Moreover, additional mutations or epigenetic factors may contribute for the differentiation into basophilic blasts.

## 1 INTRODUCTION

Acute basophilic leukaemia (ABL) is a rare subtype of acute myeloid leukaemia (AML) recognized by the 2016 World Health Organization (WHO) classification. The proposed diagnostic criteria for ABL are as follows: (a) myeloblasts + metachromatic blasts (>20%) and basophils (>40%) of nucleated bone marrow or peripheral blood cells; and (b) persistent hyperbasophilia.^1^ However, the molecular cytogenetic data available on ABL are scarce. Indeed, it remains unclear whether ABL shares a similar mutational profile with AML. Moreover, there is no established treatment strategy for ABL, highlighting the need for a better understanding of this malignancy.

Here, we diagnosed two patients with ABL based on morphology and cytology. In addition, bone marrow samples were analysed using next‐generation sequencing (NGS). This report will add new information about the mutational landscape of this rare entity.

## 2 CASE DESCRIPTION

### 2.1 Case no. 1

The patient was a 59‐year‐old Japanese man with a history of cardiac arrhythmia. One month before presentation to our institution, he was diagnosed with pneumonia and treated with tazobactam/piperacillin (TAZ/PIPC) and teicoplanin at another hospital. Three weeks before presentation, bone marrow aspiration was performed for the evaluation of leukopenia, which revealed an increased number of blasts and immature basophils. Two weeks before presentation, he was diagnosed with acute myocardial infarction (AMI), for which emergent percutaneous coronary intervention was performed. After AMI treatment, he was referred to our hospital for ABL treatment.

**FIGURE 1:**
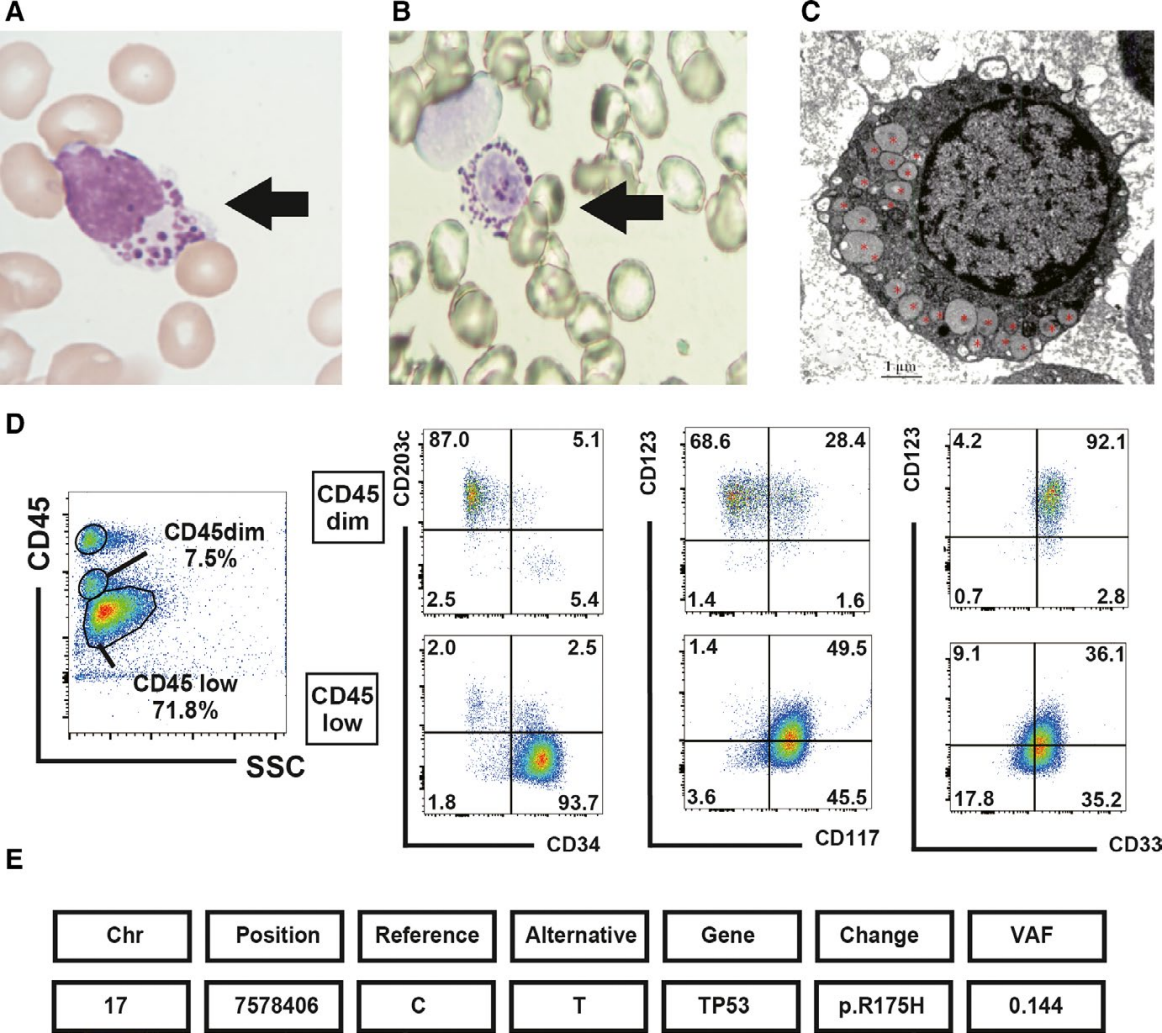
Clinical analysis of peripheral blood and bone marrow revealed an increase in blasts and basophils with TP53 mutation (Case no. 1). A, May‐Giemsa staining and B, toluidine blue staining of basophils (black arrow) in the peripheral blood (May‐Giemsa and toluidine blue staining; oil immersion lens, original magnification, ×1000). C, Electron microscopy image of basophils in the peripheral blood (red asterisks represent basophilic granules). D, Flow cytometry analysis of the bone marrow. Two tumour populations were detected: basophils (CD45dim+, CD34‐, CD203c+, CD117±, CD123+) and blasts (CD45 low, CD34+, CD203c‐, CD117+, CD123dim+), which differentiated into basophils. E, Next‐generation sequencing data

Blood examination on admission to our hospital showed a leucocyte count of 0.52 × 10^9^/L (10% blasts and 6% immature basophils). Bone marrow aspiration revealed a further increase in blasts (38.2%) and immature basophils (33.8%; Figure 1A), but no dysplasia. Toluidine blue staining was positive for immature basophils (Figure 1B). Electron microscopic examination demonstrated basophilic granules (Figure 1C). Flow cytometry detected two populations, namely blasts (CD34+, CD203c‐, CD117+, CD123dim+) and basophils (CD34‐, CD203c+, CD117±, CD123+; Figure 1D). Blasts showed basophilic differentiation. Although basophils in the bone marrow were less than 40%, his condition was consistent with ABL, based on morphology, flow cytometry and electron microscopy findings. G‐band karyotyping from the bone marrow showed a complex karyotype (42, XY, −1, −5, −7, add(7)(q11.2), −8, add(9)(p11), −10, −11, −14, der(17)t(11;17)(q13;p11.2), −18, +4mar [4/20 cells] / 43, idem, +mar [7/20 cells]/46, XY [9/20 cells]). PCR results were negative for DEK‐NUP214, and major, minor and micro‐BCR‐ABL1. NGS detected TP53 mutation (R175H; Figure 1E), 17p deletion and loss of heterozygosity of TP53.

He was administered dual histamine blockade (H_1_ and H_2_) and prednisolone to prevent symptoms due to excessive release of histamine.^1^ Induction therapy with DNR/AraC (daunorubicin 50 mg/m^2^ on days 1‐5 and cytarabine 100 mg/m^2^ on days 1‐7) was ineffective. Six weeks later, re‐induction therapy with MEC (mitoxantrone 8 mg/m^2^, etoposide 100 mg/m^2^ and cytarabine 1 g/m^2^) was administered. However, it resulted in significant inflammation and temporal cognitive impairment, possibly due to tumour lysis syndrome on day 2. Therefore, it was discontinued. He was not tolerant to high‐dose conventional chemotherapy. Four weeks later, gemtuzumab ozogamicin (GO; 10 mg/body) was administered, because the tumour cells express CD33. Following administration of GO, he developed an infusion reaction and was agitated again. He did not respond to any chemotherapeutic regimens and passed away 4 months after the initial diagnosis.

### 2.2 Case no. 2

A 72‐year‐old Japanese man with a medical history of mitral stenosis and benign prostatic hyperplasia was referred to our institute for high‐grade fever. Two weeks before presentation, he had fever and diarrhoea, which resolved simultaneously. On admission, he had high‐grade fever and hypotension. He was admitted to our hospital for suspected urosepsis as the urinalysis showed bacteriuria.

**FIGURE 2:**
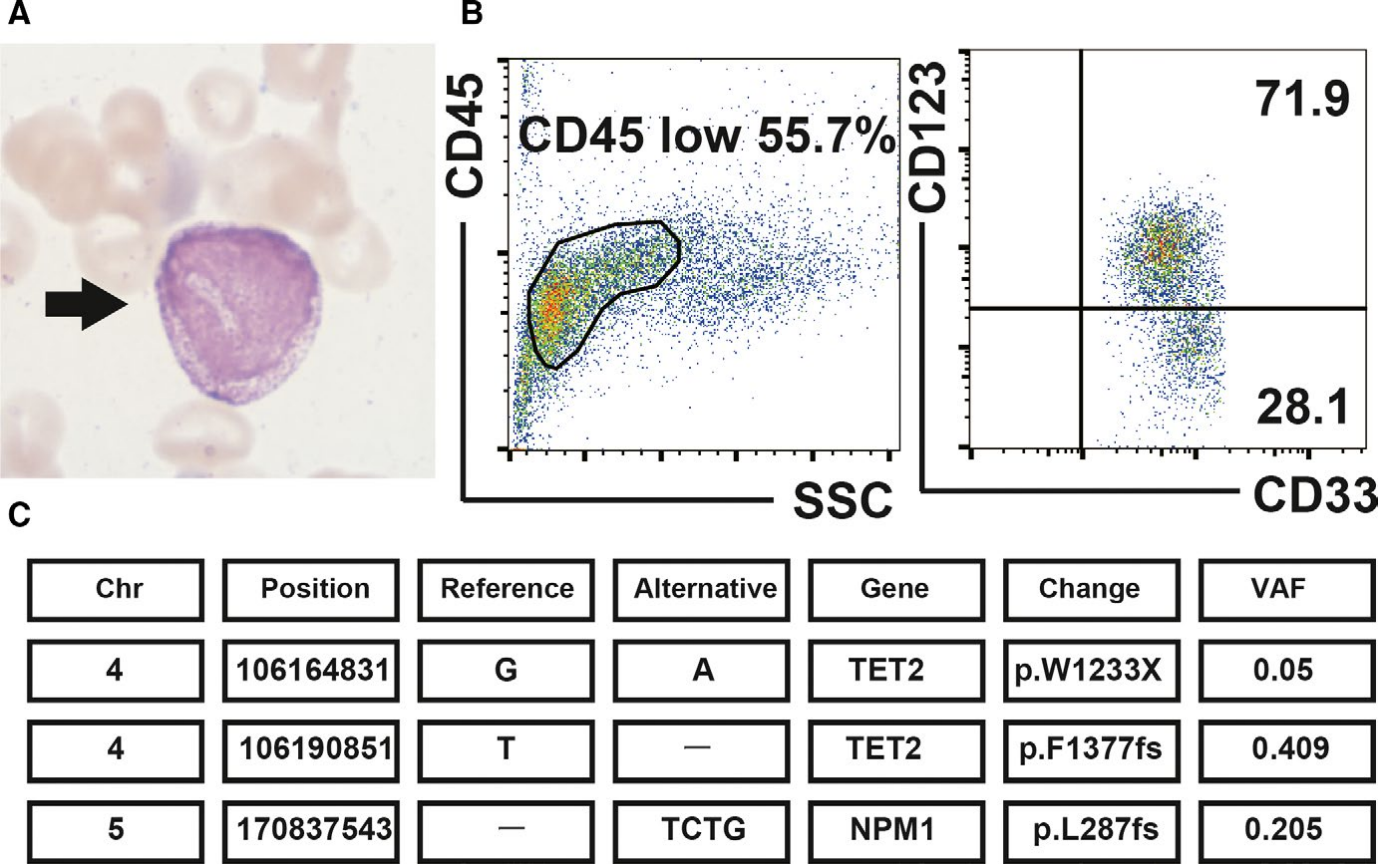
Clinical analysis of bone marrow showed an increase in blasts and basophils with TET2 and NPM1 mutations (Case no. 2). A, Basophilic granules (black arrow) were present in the bone marrow (May‐Giemsa stain, oil immersion lens, original magnification×1000). B, Flow cytometry analysis of the bone marrow: tumour cells were divided into two populations, blasts (CD33+, CD45dim+ and CD123‐) and basophils (CD33+, CD45dim+ and CD123+). C, Next‐generation sequencing data

Blood examination showed a leucocyte count of 2.62 × 10^9^/L (3% blasts and 27% immature basophils; Figure 2A) and thrombocytopenia (57 × 10^9^/L). Bone marrow aspiration revealed increased percentage of blasts (57.9%) and immature basophils (20.0%), without any dysplasia. Flow cytometry detected basophils (CD33^+^, CD45^low^, CD123^+^; Figure 2B). He was diagnosed with ABL. G‐band karyotyping showed normal karyotype (46, XY). PCR for major and minor BCR‐ABL1 was negative. NGS revealed NPM1 and TET2 mutations (Figure 2C).

For urosepsis treatment, he received meropenem and vancomycin. As hypotension persisted even after recovery from sepsis, he was administered vasopressor during induction chemotherapy. We used histamine blockers and prednisolone similar to that in Case no. 1. After induction therapy with DNR/AraC (daunorubicin 40 mg/m^2^ day 5‐7 and cytarabine 80 mg/m^2^ day 1‐7), hypotension was resolved and haematological CR was achieved at day 33. However, soon after, he had a relapse of the disease. He was refractory to subsequent chemotherapeutic regimens including mitoxantrone (MIT) monotherapy and MIT/AraC. He died 8 months after initial diagnosis.

## 3 METHODS

Data from bone marrow samples were collected using BD FACSCalibur & FACSLyric (BD Biosciences) and analysed using FlowJo version 10.

For targeted sequencing, 200 ng of genomic DNA from bone marrow was enriched for the target regions by liquid‐phase hybridization using the SureSelect custom kit (Agilent Technologies^®^). We used an in‐house panel designed to examine 390 genes that are implicated in myeloid malignancies and bone marrow failure using SureDesign (Agilent Technologies^®^). The purified library was subjected to high‐throughput sequencing analysis with Illumina HiSeq 2500 using 125 bp pair‐end mode. Sequencing reads were aligned to the human genome reference (hg19) using Burrows‐Wheeler Aligner (version 0.7.8) with default parameter settings, and annotation was conducted with Genomon pipeline 2.6.2, as previously reported.^2^ Mapping errors were resolved by visual inspection on the Integrative Genomics Viewer browser.

## 4 RESULTS AND DISCUSSION

In the present study, we presented two cases of ABL. We considered differential diagnosis of secondary cause of acute basophilic leukaemia such as blast phase of CML, AML with t(8;21) and basophilia, AML with t(6;9)(p23;q34), monocytic/monoblastic AML with basophilia, acute promyelocytic leukaemia (APL), mast cell leukaemia (MCL), MPN or MDS with basophilia. Secondary ABL was excluded based on the results of bone marrow examination, G‐banding of karyotype and detection of leukaemia fusion gene by RT‐PCR. Although a bone marrow basophil count of less than 40% in our cases did not fulfil the proposed criteria by Valent P et al^1^ there is no consensus regarding the number of basophilic blasts. Hence, we diagnosed these patients with ABL based on multiple confirmation of basophils via morphological, cytological and electron microscopic analysis. With dual histamine blockade, we were able to suppress the symptoms caused by the excessive histamine release, including shock, systemic anaphylaxis, urticaria, peptic ulceration and gastrointestinal bleeding.^3^ Multiple chemotherapeutic regimens including GO therapy were also used for Case no. 1, but none were efficacious. These cases illustrate the challenges in treating ABL.

Some reports have described cytogenetic abnormalities in ABL, such as t(X;6)(p11;q23),^4^ del(8), t(10;11)(p13;q21), del(16)(q22), t(16;17)(q22;21),^5^ t(2;6)(q23?4;p22?3), as well as del(12)(p11).^6^ However, no recurrent mutations in ABL were identified except t(X;6)(p11;q23). Case no. 1 had a complex karyotype with 17p11 translocation, whereas Case no. 2 had a normal karyotype.

To understand the underlying pathogenesis of ABL, we performed NGS in these cases. In Case #1, a *TP53* mutation (R175H) at a variant allelic frequency (VAF) of 14.4%, chromosome 17p deletion and loss of heterozygosity of *TP53* were identified. Bone marrow (38.2% of blasts and 33.8% of basophils) and G‐banding data (55% of cells harboured the 17p11 translocation) suggested that both blasts and basophils had translocation of 17p11. Considering that tumour cells with 17p10‐12 translocation led to loss of TP53,^7^ we suspected that both cell types lacked TP53 activity. Moreover, either blasts or basophils had a point mutation in *TP53* (R175H). In Case no. 2, two *TET2* mutations at VAF of 40.9% and 5% and a *NPM1* mutation at a VAF of 20.5% were identified. Both basophils and blasts seemed to carry *TET2* mutations. However, we could not determine the population harbouring the *NPM1* mutation.

Some molecular pathways may influence the phenotype and development of ABL. In four male infants with ABL, *t(X;6)(p11;q23)* and *c‐MYB–GATA1* mutations were reported. These were the first recurrent mutations of ABL^4^ and promoted basophilic differentiation by induction of *IL‐33, NGF, IL1RL1* and *NTRK1* expression.^8^ *c‐Myb* was stimulated by *p16INK4a*.^9^ In Case #2, *NPM1* through interaction with *p16INK4a* may influence c‐Myb.^10^ *C/EBPα* up‐regulation may be also associated with the development of ABL. *TET2* encodes an epigenetic modifier, which is known to act as an upstream regulator of mast cell and basophil lineage commitment. The absence of TET2 causes up‐regulation of *C/EBPα* (basophil‐specific genes).^11^ Therefore, we hypothesized that an additional *NPM1* mutation, along with a *TET2* mutation, could act synergistically to drive the leukemic transformation.^12^ *TET2* mutation combined with the epigenetic modification of *C/EBPα* may have modulated downstream molecules to induce basophil differentiation. As the relationship between *TET2, NPM1, p53* and *c‐MYB–GATA1* has not been described yet, further studies are needed to reveal the molecular link.

In conclusion, we reported two ABL cases using NGS analysis. Although mutations in these cases (*TP53*, *NPM1* and *TET2*) are common in AML, they have not been previously reported in ABL. This suggests an overlap of the mutational profiles of ABL and AML. Case no. 1 had a complex karyotype with *TP53* loss, whereas Case no. 2 had a normal karyotype with *NPM1* and *TET2* mutations, which suggests that epigenetic factors may promote differentiation into basophilic blasts. As cytogenetic and mutations of ABL are heterogeneous, further research is necessary for a better understanding on the factors that encourage basophilic differentiation in ABL.

## CONFLICT OF INTEREST

The authors declare no conflicts of interest associated with the present study.

## AUTHOR CONTRIBUTIONS

**Takuya Shimizu:** Conceptualization (lead); Writing‐original draft (lead). **Tadakazu Kondo:** Conceptualization (supporting); Project administration (lead); Supervision (lead); Writing‐review & editing (lead). **Yasuhito Nannya:** Formal analysis (lead); Methodology (lead); Project administration (supporting); Supervision (supporting); Writing‐review & editing (supporting). **Mizuki Watanabe:** Methodology (supporting); Supervision (supporting); Writing‐review & editing (supporting). **Toshio** **Kitawaki:** Formal analysis (supporting); Writing‐review & editing (supporting). **Takero Shindo:** Project administration (supporting); Supervision (supporting); Writing‐review & editing (supporting). **Masakatsu Hishizawa:** Supervision (supporting); Writing‐review & editing (supporting). **Kouhei Yamashita:** Supervision (supporting); Writing‐review & editing (supporting). **Seishi Ogawa:** Formal analysis (supporting); Funding acquisition (lead); Methodology (supporting); Project administration (supporting); Supervision (supporting); Writing‐review & editing (supporting). **Akifumi Takaori‐Kondo:** Project administration (lead); Supervision (lead); Writing‐review & editing (supporting).

## ETHICAL APPROVAL

Informed consent was obtained for using the biospecimens for research, in compliance with the Declaration of Helsinki under the protocols approved by the Ethics Committee and Institutional Review Board of Kyoto University Hospital (Ref. number: G697).

## Data Availability

The data that support the findings of this study are available from the corresponding author upon reasonable request.
